# Behavioral activation-based guided self-help treatment administered through a smartphone application: study protocol for a randomized controlled trial

**DOI:** 10.1186/1745-6215-13-62

**Published:** 2012-05-18

**Authors:** Kien Hoa Ly, Per Carlbring, Gerhard Andersson

**Affiliations:** 1 Department of Behavioral Sciences and Learning, Swedish Institute for Disability Research, Linköping University, Linköping, Sweden; 2 Department of Psychology, Umeå University, Umeå, Sweden; 3 Department of Clinical Neuroscience, Center for Psychiatry Research, Karolinska Institutet, Stockholm, Sweden

**Keywords:** Depression, Behavioral activation, Smartphone application, Cost-effectiveness, Randomized, Controlled trial

## Abstract

**Background:**

The need for cost-effective interventions for people suffering from major depressive disorders is essential. Behavioral activation is an intervention that can largely benefit from the use of new mobile technologies (for example smartphones). Therefore, developing smartphone-based behavioral activation interventions might be a way to develop cost-effective treatments for people suffering from major depressive disorders. The aim of this study will be to test the effects of a smartphone-delivered behavioral activation treatment.

**Methods:**

The study will be a randomized controlled trial with a sample size of 120 participants, with 60 patients in each group. The treatment group includes an 8-week smartphone-based behavioral activation intervention, with minimal therapist contact. The smartphone-based intervention consists of a web-based psychoeducation, and a smartphone application. There is also a back-end system where the therapist can see reports from the patients or activities being reported. In the attention control group, we will include brief online education and then recommend use of a smartphone application that is not directly aimed at depression (for example, ‘Effective meditation’). The duration of the control condition will also be 8 weeks. For ethical reasons we will give the participants in the control group access to the behavioral activation treatment following the 8-week treatment period.

**Discussions:**

We believe that this trial has at least three important implications. First, we believe that smartphones can be integrated even further into society and therefore may serve an important role in health care. Second, while behavioral activation is a psychological treatment approach for which there is empirical support, the use of a smartphone application could serve as the therapist’s prolonged arm into the daily life of the patient. Third, as we have been doing trials on guided Internet treatment for more than 10 years it is now time to move to the next generation of information technology - smartphones - which are not only relevant for Swedish conditions but also for developing countries in the world which are increasingly empowered by mobile phones with Internet connection.

**Trial registration:**

ClinicalTrials.gov NCT01463020

## Background

Major depression is expected to be the disorder with the highest disease burden in high-income countries by the year 2030 [1]. Estimated costs of depression are €177 million and €147 annually per 1 million inhabitants for major and minor depression, respectively [2]. Even if several effective psychological [3] and pharmacological treatments [4] are available for the treatment of depression, it has been shown that current treatment methods are only capable of reducing the burden of disease of depressive disorders with about one third [5]. Therefore, there is a need for cost-effective interventions that can be made available to a larger part of the population suffering from major depressive disorders. Furthermore, behavioral activation is an intervention that can largely benefit from the use of new mobile technologies (for example smartphones). One important feature of mobile technology is the possibility for the therapist to reach the patient between sessions and thus create direct incentives for behavioral activation in everyday life [6]. Therefore, developing smartphone-based behavioral activation interventions might be a way to develop a cost-effective treatment for people suffering from major depressive disorders. Another advantage with behavioral activation would be that it is more amenable to dissemination, especially via less experienced therapists and/or self-administration [7].

The efficacy of behavioral activation in the treatment of major depressive disorders has been established in a number of studies over the last four decades [8]. Furthermore, a dismantling study showed that behavioral activation can be as effective as the full cognitive behavioral therapy (CBT) treatment package [9]. In a later study, behavioral activation was found to be as effective as antidepressant medication [10], and more effective than cognitive therapy for more severely depressed patients. A series of reviews and meta-analyses also show that behavioral activation is at least as effective as full CBT packages that include both cognitive and behavioral components [11]. There are however somewhat different conceptualizations of behavioral activation, with the most recent versions covering behavioral avoidance [12]. However, all have activity scheduling as a treatment component [13]. More recent behavioral activation treatments have incorporated ingredients from Acceptance and Commitment Therapy (ACT), for example the role of values when defining treatment goals [14].

Boschen and Casey [6] summarized the advantages of using mobile phones in CBT, and the same advantages apply to smartphones: (1) mobile; (2) accepted in society in general; (3) relatively cheap (this also applies to smartphones which on average tend to be less expensive than computers); (4) they are a device with comparatively low ongoing maintenance costs; (5) they are a device already owned by a large number of people; (6) they are almost always on in that they continue to operate; (7) they are almost always connected; (8) they are programmable, meaning they are able to run novel applications software; (9) they are capable of recording media, including audio, photographs, and in many cases video, as well as being able to play or show these media to the user; (10) they are capable of interacting with the user to allow input of data using a keypad, keyboard, or touch screen; and (11) they are generally designed to be easy to use for most of the population. Moreover, mobile phones can be integrated into CBT homework, as their use attracts no attention, allowing users to interact with a handset without fear of stigma or judgment.

It is now well established that guided self-help treatment can be as effective as face-to-face psychotherapy for mild to moderate depressive symptoms [15]. Also, the treatment effects seem to persist during long-term follow-up [16]. There are, however, to our knowledge no controlled trial on Internet-delivered pure behavioral activation and no study using smartphones.

### Trial objectives and purpose

The aim of this study will be to test the effects of a smartphone-delivered behavioral activation treatment. The study is based on our previous work on guided Internet-treatment for depression [17], but in this study the treatment content will be delivered via smartphone, using a recently developed smartphone application. As waiting-list control groups are regarded as unsuitable to test the effects of treatments, we plan to have a non-specific smartphone application not dealing with depression or behavioral activation. This will probably be the first controlled study on smartphone delivered depression treatment and therefore we will begin by conducting an efficacy study [18], with participants recruited via mass media and the Internet. We expect the smartphone application to be superior to the control condition.

## Methods/design

The current study will be a randomized controlled trial with a sample size of 120 participants. The study protocol was approved by the regional Ethics Review Board in Linköping, Sweden. The protocol was approved by Ingeborg Moqvist-Lindberg and the reference number is Dnr: 2011/395-31. Written informed consent was obtained from all participants after the procedure had been fully explained.

### The intervention

An 8-week smartphone-based behavioral activation intervention with minimal therapist contact has been developed by our group. The intervention is based on our previous experiences with Internet-based depression treatment [15,16,19-23]. The smartphone-based intervention consists of a web-based psychoeducation, and a smartphone application. There is also a back-end system where the therapist can see reports from the patients or activities being reported. The concept of the intervention was built with the intention to target some of the aspects of current Internet-delivered interventions that could be improved. Although the Internet is effectively used for distributing psychological treatment programs to improve mental health [24-26], including treatment of mild to moderate major depression [27], it has been argued that Internet-based treatment applications have limitations [28,29]. One important limitation is the lack of assimilation into the user’s daily life, which is a crucial aspect of behavioral activation treatments for depression [12]. Even if paper versions of text material can be used and contact with the therapist can be handled via the Internet, these two options may not be practically feasible. Mobile phones, on the other hand, are often ubiquitous and are hence present when behaviors occur. Nowadays, access to the Internet can be facilitated by means of smartphones. Another limitation of Internet-based treatments for depression, delivered via computers, is that they tend to be text-based, often reaching close to 200 pages of reading [20]. Smartphone applications tend to be more user-friendly and condensed than regular web pages (for example the difference between a smartphone application for a newspaper and web pages for the same newspaper), which could be an attractive feature for persons with depression who easily feel fatigued and need encouragement to work through the program [30]. The latter aspect is important, as guidance appear to be crucial to prevent drop-out from guided self-help treatments [31], which was recently confirmed in a meta-analysis [15]. Finally, a challenge for modern depression treatments is to integrate information technology in the regular treatment [29]. In other words face-to-face treatments could benefit from using smartphones as an adjunct to the regular sessions, which in the case of behavioral activation treatments for depression could facilitate activity scheduling and homework, which are crucial elements of the treatment. It may also be possible to reduce the number of sessions.

A prototype of the smartphone application, together with a psychoeducation, has been tested in a pilot study (Ly KH, Dahl J, Andersson G: Development and initial evaluation of a smartphone application based on acceptance and commitment therapy, unpublished). This prototype was however not directed at depression. The results from the pilot study gave us useful information and we concluded that the intervention include some important features that could be further developed and tested. The results also showed that the smartphone application was used by some participants in situations where it was difficult to use a computer connected to the Internet, for example in the underground train, on the bus and train, and so on. More generally, this might carry implications that the platform of mobile phones does not only assimilate an intervention into a user’s daily life, but also increases the awareness of the intervention, and thus can increase adherence to the intervention. In light of these observations we believe it is feasible to proceed and test if behavioral activation can be presented in the format of a smartphone application.

### Attention control

In the attention control group, we will include brief online education and then recommend use of a smartphone application that is not directly aimed at depression (for example, ‘Effective meditation’). The duration of the control condition will also be 8 weeks. For ethical reasons we will give the participants in the control group access to the behavioral activation treatment following the 8 week treatment period.

### Sample size

We will include a total of 120 individuals. With 60 patients in each group, an effect size of Cohen’s *d* = 0.60 will be statistically significant (80% power, 5% level), which we will view as a clinically relevant difference between the two conditions.

### Referral and recruitment

Patients will be recruited via advertisement in newspapers, other media, and the Internet. Only persons who already use smartphones will be invited to participate.

### Inclusion criteria

We will include participants with an ongoing primary diagnosis of major depression of mild to moderate character. Diagnoses will be made over the telephone using the Mini-International Neuropsychiatric Interview (MINI) [32]. To administer clinical interviews via telephone has shown to give the same result as doing it face-to-face [33,34]. Medication will be allowed with the requirement that the dose is stable. Consenting participants need to be fluent in Swedish and have access to the Internet as well as a smartphone.

### Exclusion criteria

Persons with suicidal intent and patients for whom the depression is directly linked to another major primary psychiatric disorder (for example, psychosis or bipolar disorder) will be excluded and referred to other treatment resources. In addition, people with a history of misuse of alcohol or drugs will be excluded.

### Informed consent

Participation will require a written informed consent.

### Withdrawal

Participants can at any time withdraw from the treatment or the study without specifying the reason.

### Safety monitoring and reporting

We will adhere to the Swedish Personal Data Act. No sensitive data will be saved on a computer in which the person providing data can be identified. All Internet and smartphone activities will be highly secure, with encrypted information as well as no contact outside of a closed contact system. If participants decline further participation, the data will be removed.

### Outcome measures

The primary outcome measures will be self-assessed depression using the Beck Depression Inventory (BDI-II) [35] and the Patient Health Questionnaire-9 (PHQ-9) [36]. PHQ-9 consists of nine items, each scored 0–3 and with a total score range from 0 to 27. PHQ-9 shows a sensitivity of 0.77 (0.71–0.84) and a specificity of 0.94 (0.90–0.97) [37]. BDI was constructed to assess the degree of depression in adolescents and adults, and consists of 21 questions [38]. The BDI has been extensively validated in various studies. A review of the BDI’s internal consistency estimates yielded a mean coefficient alpha of 0.86 for psychiatric patients and 0.81 for non-psychiatric subjects [39]. For psychiatric patients the test-retest reliability has been reported to range from 0.48 to 0.86, whereas the coefficients for non-psychiatric subjects ranged from 0.60 to 0.83 [39]. We will also administer the MINI [32]. This will be done via telephone to make participation possible for persons who live in all regions of Sweden. Moreover, an independent (blind) rating of clinical global impression-improvement scale (CGI) [40] will be performed. Hence, all patients will be interviewed again after the treatment period. All self-report measures will be administered via the Internet, which will facilitate 1-year and 2-year follow-ups. According to Thorndike *et al*. [41], self-report measures administrated via the Internet show good validity. We estimate a drop-out from completing the post-treatment measures of approximately 30%. This would lead to a reduced sample size of 84, which will maintain the estimated power. While the cross-over design will prohibit conclusions regarding specific long-term effects, the long-term effect of the new treatment format (that is smartphones) is not yet investigated, making the follow-up informative in the case both treatments are ineffective. Moreover, switching treatment will be optional and we will be able to investigate subgroups that only complete one of the treatments. The main outcome of interest in the study is if smartphone-enabled behavioral activation is superior to attention control (use of smartphone with no reference to depression or behavioral activation). We expect a moderate difference on the main outcome measures of depression. We also expect the number of responders (defined as a decrease according to Jacobson on the BDI-II [35] and PHQ-9 [36] and remission to lower levels of symptoms on the MINI-mood disorders section [32]) to be larger in the behavioral activation arm.

### Moderators and mediators

As secondary outcome we will include the Beck Anxiety Inventory (BAI) [42], the Quality of Life Inventory (QOLI) [43] and Acceptance and Action Questionnaire (AAQ-II) [44]. The BAI is a 21-item self-report inventory for measuring the severity of anxiety in psychiatric populations [42]. De Beurs *et al.*[45] showed that the BAI has high internal consistency in panic patients (Cronbach’s a = 0.92) and good test-retest reliability (0.83). The QOLI consists of 16 areas rated by the subject concerning importance and satisfaction. The instrument’s internal consistency is high, between a = 0.77 and 0.89, and the 1 month test-retest reliability lies between r = 0.80 and 0.91 [43]. The AAQ-II is a 10-item, 7-point Likert scale that assesses psychological flexibility - a term for the ability to accept unwanted internal experiences and engage in valued actions. The AAQ II has shown good reliability (test-retest reliability = 0.81–0.87) and validity [44].

Other measures will be included as potential predictors of outcome such as education, number of previous episodes, and so on. Finally, we will include the Trimbos and Institute of Medical Technology Assessment Cost Questionnaire for Psychiatry (TIC-P [46]), which is a questionnaire designed to measure health related costs. Cost-effectiveness studies also often involved a measure called the EQ-5D [47]. The study will follow the guidelines given in the CONSORT statement [48,49], with the exception of double-blinding, which is not possible in psychotherapy studies. Other features such as intention-to-treat (mixed models approach [50]) and statistical power calculations will be adhered to. The theoretical rationale for smartphone supported behavioral activation is the observation that activity is related to depressive symptoms [8] and that behavioral change may be facilitated by the increased adherence provided by the smartphone.

### Statistical analysis

An intention-to-treat design will be used when analyzing post-treatment data and data collected at 1-year follow-up. Between-group changes at post-treatment and at 1-year follow-up will be calculated using analyses of covariance (ANCOVAs), with pre-treatment scores as the covariate. Effect sizes between and within the two groups will be calculated with Cohen’s d computed with the pooled standard deviation.

## Discussion

We believe that this trial has at least three important implications. First, we believe that smartphones can be integrated even further into society and therefore may serve an important role in healthcare. It has been suggested that mobile phones have become an important part of our everyday lives, and that they already are providing us with several services, for example phone calls, text and multimedia messages, cameras, music, calendar and the Internet. Therefore, adding applications to facilitate health into the platform may be perceived by the user to be a natural step, and thus can be reached out to more people. In fact, there are already several smartphone applications aimed at mental health, but unfortunately a vast majority have not been tested in research and may be limited in terms of content. Should we be able to show that mild to moderate depression can be treated effectively by means of a supported smartphone application, it is highly possible that this will be followed by applications for other health problems.

Second, while behavioral activation is a psychological treatment approach for which there is empirical support, the use of a smartphone application could serve as the therapist’s prolonged arm into the daily life of the patient. Since the theoretical understanding of the mechanisms behind behavioral activation suggests that behaviors should be immediately reinforced when they occur, our smartphone application may lead to improved compliance compared to our earlier Internet treatments.

Finally, from a psychiatric research point of view our group is probably among the most active research groups in Sweden when it comes to conducting randomized controlled trials on psychiatric conditions. As we have been doing trials on guided Internet treatment for more than 10 years it is now time to move to the next generation of information technology - smartphones - which are not only relevant for Swedish conditions but also for developing countries in the world which are increasingly empowered by mobile phones with Internet connection.

## Trial status

Active, not recruiting. However, when the manuscript was submitted to the journal the study was in recruitment.

## Competing interests

A related version of the application is currently developed for the open market.

## Authors’ contributions

KHL is the project manager and has developed the application. KHL will also participate in the drafting of treatment manuals, perform the treatments, and participate in analysis and interpretation of data. GA participated in the conception of the study and its design. GA will also participate in the drafting of treatment manuals, analysis and interpretation of data, and perform statistical analysis. PC participated in the conception of the study and its design. KHLand GA drafted the current manuscript. PC participated in revision of the current manuscript. All authors read and approved the final manuscript.

## Authors’ information

KHL is a PhD student in Clinical Psychology at Linköping University in the Department of Behavioural Sciences and Learning. GA is professor of Clinical Psychology at Linköping University in the Department of Behavioural Sciences and Learning. GA also holds a position as researcher at the Karolinska Institute, Stockholm in the Department of Clinical Neuroscience, Psychiatry (section for Internet psychiatry). GA is also editor for *Cognitive Behaviour Therapy*, *BMC Psychiatry* and section editor for *Scandinavian Journal of Psychology*. GA is also founding member of the International Society for Research on Internet Interventions and member of the Swedish Institute for Disability Research (SIDR). PC is Professor at the Department of Psychology at Umeå University. PC is also editor-in-chief for *Cognitive Behaviour Therapy. Additionally*, PC is the secretary of the non-profit interdisciplinary organization International Society for Research on Internet Interventions.
